# Proceedings of the Pathological Society of Philadelphia

**Published:** 1859-05

**Authors:** 


ART. III.—*Proceedings of the Pathological Society of Philadelphia.*

Reported by the SECRETARY.

WEDNESDAY EVENING, February 9th, 1859.

Vice-President, DR. STILLÉ, in the Chair.

*Pseudo-Membranous Laryngitis in an Adult.*—DR. ALBERT H. SMITH presented a specimen of pseudo-membranous laryngitis, in which the false membrane had been moulded into a cast of the trachea and ramifications of the bronchi.

Louisa Jones, aged twenty-two, single, native of Canada, domestic, was admitted into the Pennsylvania Hospital, December 23, 1858, with chronic bronchitis and amenorrhœa. She had had a cough for about three months, not very severe, and not increasing; she suffered with violent headaches, and had not menstruated for four months. When admitted, she complained of pain in the chest over the sternum; of headache; coughed considerably, and expectorated tenacious mucus; had mucous râles in the lower posterior part of both lungs, with sonorous and sibilant râles above and in front; percussion was normal over the whole chest. There was no marked emaciation; her appetite was tolerably good. She slept badly, but had no night-sweats.

She was put upon treatment for the bronchial affection, and also in reference to the restoration of her menses. She improved considerably in her general health; increased in flesh, her appetite improved, and the chest symptoms diminished, though there still remained some sonorous and mucous râles.

On the nineteenth of January, she complained of great soreness of her throat, with difficulty of swallowing, which had been coming on for several days; but as she had frequently had attacks of the same kind, she thought it would pass away, and did not mention it. On examining the throat I found considerable enlargement of both tonsils, the right being much the larger. The usual means were taken to reduce the inflammation: the tonsillitis appeared to be running through its regular course. The day following this attack she menstruated. On the twenty-first, the swelling had increased; she swallowed with great difficulty, and at times had dyspnœa, although not violent. The tonsils almost occluded the pharynx and fauces; they were still hard. A lancet was plunged into them, but no pus escaped. The chest symptoms were in no respect changed. On the twenty-third, the difficulty of breathing had increased; the tonsils completely closed the fauces. A very deep incision was made with a scalpel into the substance of each tonsil; a free flow of blood took place, but no pus. There was still no increase of the physical signs in the chest, so far as could be detected on careful examination. In the evening, she told me she felt much better; her breathing was much more free, and she had swallowed some soup. I did not at this time examine her lungs, as I had thus far had no reason to suspect anything more than a severe tonsillitis. About three hours afterwards, while I was absent for a short time from the hospital, my colleague, Dr. Hutchinson, was called to her with the statement that she was suffocating; he went, but before he reached her she had expired. Nothing could exceed my surprise, on returning an hour later, than to be informed of her death.

Autopsy, made eighteen hours after death.—The trachea and œsophagus were cut across the middle and carefully dissected up as far as the posterior nares; the tongue and pharynx were removed with the tonsils. These last were found enlarged so much as to close almost entirely the air-passages, the right being as large as a walnut. Lining the pharynx posteriorly to the tonsils, and resting upon them, were large flakes of membranous exudation, at least one-sixteenth of an inch in thickness; they could, however, be removed with great facility. Passing downward, we found the upper surface of the epiglottis invested with the same exudation, and a band of firm membrane passing from it to the pharynx posteriorly, and preventing it from closing. The under surface was also coated with exudation, and on opening the larnyx and trachea, we noticed a continuous membrane investing the whole of the larynx, becoming in the trachea a perfect tube-cast that could be readily separated from the mucous membrane, which was highly injected. On further examination of the portion of trachea which had been cut off, this cast was found to extend, without any interruption or break, into the secondary ramifications of the bronchi.

The lungs were entirely free from tuberculous deposit, but slightly emphysematous above; the other organs of the body were perfectly healthy.

*Case of Sanguineous Apoplexy of Ventricles and Subarachnoid Space.*—DR. ALBERT H. SMITH presented a specimen of apoplexy.

Ann Jones, aged seventy, colored, native of Philadelphia, cook, was conveyed to the Pennsylvania Hospital, on the afternoon of January 29, 1859, by two policemen, who had found her in the street in a state of insensibility. As was afterwards learned, she was a woman of remarkably vigorous constitution, her friends having no recollection of her ever having been sick; she was a widow, with eight children living; had been in the habit of going out as cook, and left the house where she boarded on the morning before her attack, in apparently perfect health. Nothing further could be ascertained of her until she fell suddenly while walking in the street, and was immediately brought into the hospital. She was a woman of strictly temperate habits in every respect.

When admitted, she was lying motionless, except the quick heaving of her chest; completely comatose, and not giving the least indication of consciousness on being shaken or loudly called. Her skin was cool and dry; her pulse forty, very irregular, full but compressible, occasionally intermittent, with constant variation in its force; respiration forty-five per minute, regular in rhythm. Her pupils were contracted to a point, and were entirely insensible to light. There was no strabismus, nor facial distortion; and not the least stertor. Her breath had a peculiar vegetable odor, somewhat narcotic.

Her stomach was thoroughly washed out—an emetic of sulphate of zinc acted promptly; a purgative injection also operated readily; but she remained in the same condition as before. While being cupped on the back of the neck, she gaped, but gave no further evidence of consciousness or muscular power.

On the following day she was still in a profound coma; not the least sensibility or consciousness: while being bled from the temporal artery, she gaped a second time. Her pulse was sixty, full, but weak, quite regular; respiration thirty, regular; action of heart normal; no symptoms of pulmonary affection. After the abstraction of about six ounces of blood, her pulse rose very considerably in strength, and until eighteen ounces were taken continued so, when it fell to a natural condition. Her rectum and bladder required artifical evacuation. On the third day, there was no change, except that once, on being pinched, she moved all of her limbs and rolled slightly over; she also swallowed some beef-tea poured into her mouth; the pulse, respiration, and the general symptoms remained unaltered. On the fourth day, there was some stertor; spastic rigidity of the flexors of the arm and hand on both sides; no other irregular muscular action. Pulse sixty, and natural; respiration also natural. She had the appearance of one enjoying a tranquil sleep.

On the fifth day, there was no change.

On the sixth, her condition was greatly altered; her skin was cold and clammy; her pulse 120, very weak; stertor marked; froth about her lips; she had involuntary evacuations from her bowels and bladder. After I saw her in the morning she gradually sank until three o'clock P.M., when she died.

Autopsy.—On removing the calvaria, the dura mater was found quite healthy; there was no effusion, either sanguineous or serous, in the arachnoid cavity. The arachnoid was itself in places opake and thickened; the subarachnoid spaces, in the sulci and at the base of the brain, were filled with dark liquid blood, amounting in quantity to about two ounces; the pia mater was greatly injected. On cutting into the substance of the brain it was found firm and healthy. Both ventricles were distended with coagula, filling completely their cornua; the two ventricles communicated, the septum lucidum being ruptured. There was great injection of the surfaces of the ventricles, but no lesion of either thalamus or corpus striatum. There was no other abnormal condition of the brain.

The other organs gave no evidence of disease; a moderate amount of atheroma in the ascending aorta was observable.

*Cancer of the Hand.*—DR. LENOX HODGE presented a cancer of hand, which was amputated by Dr. Peace, before the class at the Pennsylvania Hospital.

The patient from whom it was removed is a man of about fifty-five years of age. He states that about seven years ago he noticed what he supposed to be a wart, growing on the back of his right hand, at the base of the first finger. It gradually increased for two years, when it had become half an inch in height and as large as the end of his finger. He applied to a surgeon, and had it removed. The wound healed in three or four weeks, and remained well for twelve months, when the lower part of the cicatrix began to ulcerate. The ulcer continued to spread, he says, under all applications, till it extended, on the back of the hand, from the third finger to the thumb, and around on the palm beneath the first finger.

The history thus appears to be that of epithelial cancer, and its microscopical examination corresponds with what Mr. Paget describes as belonging to that form of cancer.

---

WEDNESDAY EVENING, February 23, 1859.

Vice-President, DR. STILLÉ, in the Chair.

DR. HALL exhibited a specimen of tubercular deposition in the lung, following typhoid fever.

DR. LA ROCHE stated that such results, following low fevers, were more frequent in the negro than in the white. During an epidemic of typhoid fever at the Walnut Street Jail and Almshouse, many years ago, it was found that in all negroes dying of the fever, tubercular deposits existed in the lung.

DR. KELLER spoke of the difficulty of distinguishing cases of acute phthisis from typhoid fever. As a diagnostic sign he alluded to the different smell. The peculiar odor of typhoid fever has been noticed by different observers. Several years ago his attention was arrested by the odor of patients who suffered from acute tuberculosis. He has since found this odor almost invariably present. Its character is very different from that of typhoid fever, yet it cannot be exactly described. It is very peculiar and very constant.

*Cancer of the Bladder and Other Organs.*—DR. DARBY presented, through DR. WOODWARD, several specimens of internal cancer.

CASE I. *Cancer of the Bladder; Single Kidney.*—Nancy K., aged sixty, born in Ireland, married, and moderate in habits, was admitted into the Philadelphia Hospital on November 11th, 1858.

The history given by the patient was rather complicated, yet the following points are reliable: She was admitted into the hospital five years previously, where she remained for a year or longer, suffering from an affection of the bladder and kidneys. She was exhibited to the clinical class at that time, and treated for hæmaturia. After leaving the hospital in the same condition in which she entered it, she resided in the city, without any abatement of symptoms, and was readmitted in almost a moribund condition. Patient complained of intense burning in hypogastric region, great thirst, and disorder of the whole digestive organs. The expression of countenance was marked by intense suffering; the skin was dry, with a dull-yellow hue; tongue thickly coated; pulse weak, soft, and thread-like, with ninety pulsations to the minute. She passed constantly from the sexual organs a thick, dark-colored fetid fluid, and in such quantities as to cause a change of clothing three or four times daily. Her bowels had not been moved for ten days prior to her entrance, and then by the use of a purgative. The stomach was irritable, scarcely retaining the lightest farinaceous articles. Above the pubes there was prominence which was hard and resisting to the touch.

A vaginal examination was attempted, but from the pain given, and weakness of the patient, was discontinued. The symptoms, taken together, induced me to diagnosticate cancer of the uterus and bladder.

The treatment was palliative and supporting, anodynes, milk-punch, with lime-water, cold medicated cloths applied over the pubic region, and the bowels moved by enemata. In twelve days after being admitted, she died. In eighteen hours after death, a post-mortem was made, which corroborated the diagnosis.

Post-mortem examination.—The lungs, at two points immediately opposite, one on the right, the other on the left lung, presented small, yellowish spots the size of a pea, which exuded, when cut, a thick fluid, having, when placed under the microscope, the usual cancer-cells; the remaining part of the lungs was healthy. The heart, stomach, liver, spleen, pancreas, and kidney were healthy. There was only one kidney with its supra-renal capsule; no trace whatever existed of either of these organs on the left side; and no vessels running from the aorta, which was taken out as proof that these organs did not exist on the left side. The intestines adhered together from inflammatory action produced by pressure of the cancerous mass, which filled the whole pelvic cavity, as well as being situated on the lower lumbar vertebræ The bladder was thickened, and a massive vascular papillary growth, springing from its walls, projected into the cavity. The lymphatic glands of the mesentery were converted into soft pulpy tumors of moderate size, which were in some parts distinct, in others less so.

The vesical walls, the papillary growth in the bladder, and the enlarged mesenteric lymphatics, all more or less soft and brain-like to the eye, permitted a thick creamy juice to exude, when cut surfaces were scraped. In this juice the following elements were detected, by Dr. Woodward, who kindly examined it:—

1. Innumerable granules, chiefly Aschersonian vesicles, of variable size.

2. Free nuclei in comparatively small numbers, of great size and dimly granular, with one, two, or three nucleoli.

3. Innumerable cells of varied shape, containing one, two, or more nuclei, already described. These cells contained often a number of large Aschersonian vesicles, and were more granular than the nuclei.

These elements could be plentifully obtained from all parts of the morbid masses, and, taken in connection with the general appearances of the new formations, justify us in regarding the case as one of medullary cancer.

CASE II. *Cancer of the Uterus.*—Amanda C., a Philadelphian by birth, married and temperate, was admitted into the Philadelphia Hospital, November 12, 1858. Patient was forty-six years of age.

She had been treated in several hospitals in this city, and had been in this house three or four times, in different wards, in the last four years. This case was transferred from the venereal ward to the surgical, in January, 1859. The history of the case is as follows:—

For five or more years she had been suffering from uterine complaints, and had several times, in hospitals, been treated for affections of the bladder and womb, with only temporary relief. Several months previous to her last entering this hospital, she had passed from her uterus constantly a dark fetid liquid, which caused much annoyance on account of odor, quantity, and pain. She was unable to work, from pain in stooping, and could not sleep unless she took a dose of laudanum before retiring. The general aspect of the patient was anæmic, with a dirty-yellow hue of the whole surface and also of the conjunctiva. The expression of face indicated mental anxiety and pain, the skin was dry, the pulse weak, pulsating over eighty to the minute, tongue coated, and the alimentary canal deranged in function. The stomach was in such a state as to permit little to remain on it, and the bowels so torpid, as only to be roused by purgatives or enemata. Soon after she entered the ward, a per-vaginam examination was made, which told the same tale that she had given; and, taking all the symptoms, and recollecting at the same time the similarity in almost every respect to the case which had but a short time since died, I again diagnosticated cancer. A similar treatment was adopted, and in a short time a similar result took place. Prior to her death, she suffered much from abdominal dropsy; and when, thirty hours after death, I made a post-mortem examination, more than two gallons of fluid were collected. This effusion was produced by the pressure of the cancerous masses of the abdominal viscera upon the veins. These cancerous masses, wherever found, presented the usual characteristics of the nuclei and cells, so often described.

The liver was in a state of fatty degeneration, and the nodules in it exhibited the usual anatomy of cancer. The kidneys were also in a state of fatty degeneration. The pancreas appeared normal; so did the spleen. The mesenteric lymphatics were cancerous. The uterus was the same, and so broken down in its structure, as to tear when being taken from the cancerous mass, which filled the whole pelvic cavity, and which had caused, by its pressure, the fæces to be retained in hardened masses, and gangrene of parts of the colon. The stomach was much inflamed from pressure upon it; this accounts for its highly irritable state. The left lung was highly congested, particularly the lower lobe; and immediately beneath the pleura, in connection with the fibrous layer of that membrane, were a number of thin bodies, one-fourth to three-fourths of an inch in diameter, one-eighth to one-fourth in thickness, around which the pleura was slightly puckered. These bodies presented the anatomy of cancer. The right lung appeared healthy, and so did the pleura; yet the touch revealed nodules varying in size, the longest about one-fourth of an inch in diameter. These nodules were soft and pultaceous, and exhibited fat granules and angular nuclei, undistinguishable from those of tubercle.

DR. WOODWARD made some remarks in connection with these specimens. The fatty degeneration observable in the liver is not an unusual accompaniment of cancer. To this fatty degeneration may be owing the peculiar cancerous color. The cancerous cachexia is not constant. It is very frequently absent in external cancers. Indeed, it does not appear until a few weeks prior to the death of the patient. It is most marked in internal cancers, especially those of the liver. It is, therefore, not the cancer itself, but the fatty state of the liver, which occasions the peculiar color which is supposed to indicate the cancerous cachexia.

*Kidneys of Different Size.*—DR. LENOX HODGE exhibited two unsymmetrical kidneys.

J. W., aged forty-five years, was admitted into the Pennsylvania Hospital with a fracture at the base of the skull. He died on the third day after his accident. In making the post-mortem examination, we found the right kidney to be double the normal size, while the left one was no larger than a Lima bean. Both kidneys were in their natural positions; both ureters were normal in position and size; and tubular throughout their whole extent. Every part of the body was well developed and symmetrical, except the kidneys. The urinary secretion was carried on as if both kidneys were in perfect action; there was apparently no difference, either in quantity or quality.

The small kidney was referred for microscopical examination to a committee.

*Aneurism of the Arch of the Aorta.*—Dr. Brinton exhibited a specimen of aneurism of the aorta, presented by Dr. Hay, United States Navy.

The patient, a Swede, aged sixty-four, had been admitted into the United States Naval Asylum on the eighth of December last. A large pulsating tumor, which had existed for sixteen years, was observed in the supra-sternal fossa, overlapped on either side by the edge of sterno-cleido-mastoid muscle. The patient, who was weak and prostrated, complained of pain in the tumor, and suffered much from vertigo and repeated asthmatic attacks. During the latter part of December his symptoms became much less marked, and he appeared comparatively comfortable. In the early part of January the pain in the tumor returned, and became aggravated, so as to produce a violent burning sensation. Constant and violent vomiting ensued; coming on often without effort on the part of the patient; the substances vomited, were frequently mixed with blood.

During the last days of January these symptoms increased, and the man suffered from repeated attacks of vertigo. He then gradually sank, and died from exhaustion on the eighth of February.

On post-mortem examination, a large aneurism of the arch of the aorta was revealed; the arteria innominata was much enlarged and sacculated; the calibre of the left carotid and subclavian was increased, and the walls of these vessels were greatly thickened. The dilation of the aorta extended down to the diaphragm, a portion of the tumor projecting into the abdominal cavity. At this point the bodies of the vertebræ had undergone partial absorption.

Considerable pressure had been exerted by the tumor upon the œsophagus; and the pneumogastric nerve of the left side had been flattened out for an inch in extent, so as to render it probable that its function had been much interfered with.

Dr. Brinton drew the attention of the Society to this fact, and referred to a case of section of this nerve, during the performance of the operation of extirpation of the parotid gland by Dr. M'Clellan, in which no injurious consequences resulted.

